# ColPortal, an integrative multiomic platform for analysing epigenetic interactions in colorectal cancer

**DOI:** 10.1038/s41597-019-0198-z

**Published:** 2019-10-31

**Authors:** Angel Esteban-Gil, Fernando Pérez-Sanz, José García-Solano, Begoña Alburquerque-González, María Antonia Parreño-González, María del Carmen Legaz-García, Jesualdo Tomás Fernández-Breis, Edith Rodriguez-Braun, Paola Pimentel, Anne Tuomisto, Markus Mäkinen, Ondrej Slaby, Pablo Conesa-Zamora

**Affiliations:** 1 Biomedical Informatics & Bioinformatics Platform, Institute for Biomedical Research of Murcia (IMIB)/Foundation for Healthcare Training & Research of the Region of Murcia (FFIS), Calle Luis Fontes Pagán 9, 30003 Murcia, Spain; 2 Department of Pathology, Santa Lucía General University Hospital (HGUSL), Calle Mezquita sn, 30202 Cartagena, Spain; 3 Department of Histology and Pathology, Faculty of Life Sciences, Catholic University of Murcia (UCAM), Murcia, Spain; 4 Research Group on Molecular Pathology and Pharmacogenetics, Institute for Biomedical Research of Murcia (IMIB), Calle Mezquita sn, 30202 Cartagena, Spain; 5 Departamento de Informática y Sistemas, Universidad de Murcia, IMIB-Arrixaca, 30100 Murcia, Spain; 6 Department of Oncology, HGUSL, Calle Mezquita sn, 30202 Cartagena, Spain; 7 Department of Pathology, University of Oulu, Aapistie, 9, 90014 Oulu, Finland; 8 Central European Institute of Technology, Masaryk University/Department of Comprehensive Cancer Care, Masaryk Memorial Cancer Institute, Faculty of Medicine, Masaryk University, Kamenice 753/5, 625 00 Brno, Czech Republic; 9 Department of Laboratory Medicine, HGUSL, Cartagena, Spain

**Keywords:** Diagnostic markers, Colon cancer

## Abstract

Colorectal cancer (CRC) is the third leading cause of cancer mortality worldwide. Different pathological pathways and molecular drivers have been described and some of the associated markers are used to select effective anti-neoplastic therapy. More recent evidence points to a causal role of microbiota and altered microRNA expression in CRC carcinogenesis, but their relationship with pathological drivers or molecular phenotypes is not clearly established. Joint analysis of clinical and omics data can help clarify such relations. We present ColPortal, a platform that integrates transcriptomic, microtranscriptomic, methylomic and microbiota data of patients with colorectal cancer. ColPortal also includes detailed information of histological features and digital histological slides from the study cases, since histology is a morphological manifestation of a complex molecular change. The current cohort consists of Caucasian patients from Europe. For each patient, demographic information, location, histology, tumor staging, tissue prognostic factors, molecular biomarker status and clinical outcomes are integrated with omics data. ColPortal allows one to perform multiomics analyses for groups of patients selected by their clinical data.

## Abstract (summary)

**Table:** 

Measurement(s)	miRNA • methylation • clinical history • histology • transcription profiling assay • microbiome
Technology Type(s)	DNA sequencing • clinical monitoring • RNA sequencing • amplicon sequencing • ex vivo photography with digital image analysis • methylation profiling by array
Factor Type(s)	tumor status
Sample Characteristic - Organism	Homo sapiens

Machine-accessible metadata file describing the reported data: 10.6084/m9.figshare.9785795

## Background & Summary

Colorectal cancer (CRC) is the second most commonly diagnosed cancer in Europe and the third leading cause of death worldwide. In 2012, 1.4 million new cases were diagnosed and 694,000 deaths were reported^1^. CRC development is a result of combined genetic and lifestyle factors, in which diet, immune system status and microbiota seem to be involved. Interestingly, genetic and epigenetic alterations and histopathological and clinical features are different in proximal and distal CRCs^2^. The adenoma-carcinoma sequence of CRC carcinogenesis is typically characterized by distal colon location, chromosomal instability and microsatellite stability (MSS), leading, ultimately, to the development of conventional colorectal carcinoma (CC) accounting for around 80% of CRC^3^. However, less is known about the serrated carcinogenic pathway although it has been associated with proximal colon location, female gender and having high-level of microsatellite instability (MSI-H)^2,4^. It is assumed that serrated adenocarcinoma (SAC) and CRC showing histological and molecular features of MSI-H (hmMSI-H)^2,5^ are both end-points of this serrated pathway^6^. SAC has been recognized in the latest WHO classification of tumors of the digestive system as a new subtype of colorectal cancer (CRC)^7^, accounting for 7.5–8.7% of all CRCs^8,9^. SAC has been shown to have a worse prognosis than CC^9^, partly due to a higher frequency of adverse histological and molecular features at the invasive front such as high grade tumor budding, infiltrating tumor growth and weak peritumoural lymphocyte response^10^. On the contrary, hmMSI-H is characterized by the occurrence of a Crohn-like reaction and peri- and intra-tumoral infiltrates^5^. Moreover, transcriptomic and methylomic studies carried out by our group using arrays have demonstrated that SAC differ from CC and hmMSI-H in gene expression and CpG methylation profiles^11–13^. More recently, growing evidence points to a causative role of bacteria in the colorectal carcinogenic process^14,15^ thus suggesting that different histological subtypes and locations of CRC would be explained by different bacteria populations whose relative composition depends on host diet and the anatomical portion of the colon^16^. In fact, it has been reported that serrated colorectal pathway and MSI status are associated with the presence of particular bacteria^17,18^. Whilst the hypermethylation of promotor regions which cause epigenetic silencing of tumor suppressor genes is clearly understood as an oncogenic mechanism^19^, an intriguing issue is how global hypomethylation at CpG sites outside gene promoters can contribute to cancer development. Growing evidence suggests that the expression of non-coding RNAs, such as microRNA, is activated thus favoring tumor development and progression. In addition, the histological image is a complex manifestation of all these molecular alterations, especially at the tumor invasive front where the cancer cells interact with stromal and immune system cells. As CRC is diagnosed by histological examination, an important aspect of analysing high-throughput data is to consider the context of the microscopic morphology of the tissue.

By incorporating unpublished datasets on microbiome and microtranscriptome from our CRC series, our group has integrated this information with published methylome and transcriptome data^11–13^ from the same cases to present here ColPortal, a free online platform to analyse multiomics and clinico-pathological features of CRC including digital histological images to help researchers unveil some pending questions, as explained above, related to colorectal carcinogenesis.

## Methods

### Web platform

Our proposal is based on our previous results and experience working with biomedical data^20,21^. ColPortal (https://colportal.imib.es) aims at providing an open web platform where users can download, visualize and analyse clinical and omic data in an integrated way. One of the main features of ColPortal is the possibility of making integrated analyses in real time. ColPortal has been developed using Java and R technologies and deployed in a HP Proliant DL360 G9 server with two Intel xeon processors with 12 cores (hyperthreading) and 256 GB RAM.

**Fig. 1:**
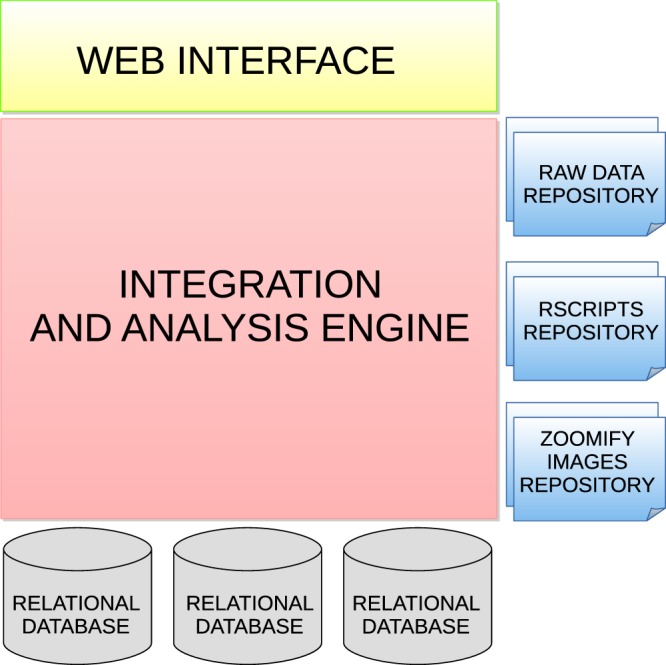
ColPortal Technological Architecture.

Figure 1 shows the technological architecture of ColPortal, whose main modules are described below:

- **Web Interface**. This is a web application developed using Java Server Faces technologies. Web standards (HTML, CSS) have been used to display the data. This means that any user can use ColPortal without requiring any third party software.
- **Integration and Analysis Engine**. This module allows the communication between the web interface and the several data sources. The clinical data are used for generating the cohorts for the personalized analyses. In the current implementation, methylome data is used in every analysis, because this is the omics type for which we have the largest amount of samples. Methylome is actually the omic data that requires more computational time. For example, the analysis of normal/tumoral cases may take up to 20 minutes using the server described above. Consequently, methylome data have been pre-analysed to ensure that real-time multiomics analyses can be executed in ColPortal. Furthermore, this module is able to recover the data filtered by the user, perform statistical analyses using R libraries, and load the results in an in-memory database, so the data can be used by visualization methods or reused in new studies.
- **Relational Database**. A MySQL relational database contains information about clinical cases, molecular features, tissue images and the links to the omics files for the integrative analysis.
- **Linked Data Database**. We use Virtuoso as a Linked Data database. In this case, all the data generated in differential methylation analysis are stored in RDF to facilitate the exploitation of this large dataset (450,000 probes by clinical case).
- **In-memory Database**. All the data results from analysis generated in real time are stored in an Sqlite database. This database engine allows the use of an in-memory temporary database. This feature is very interesting to increase performance.
- **RAW data repository**. The entire raw dataset from the different omics is stored in a server folder. The corresponding paths are stored together with the clinical cases in the relational database.
- **R scripts repository**. The analysis of omics data is supported by R scripts. Parameterized R scripts are stored in a folder of the server. When the user selects the information to be analysed, we use the R scripts as templates to generate the concrete code.
- **Zoom-in images repository**. All the images that are zoomed in the visualization tool are stored in a server folder and indexed with the clinical cases in the relational database.

### Types of analysis

Currently, ColPortal allows the following types of analysis:

- **Multiple Correspondence Analysis (MCA)**. FactoMineR package is used for multiple correspondence analysis^22^. Factoextra package^23^ is used to visualize the results of MCA analyses.
- **Differential gene and miRNA expression analysis**. Limma from Bioconductor is used for differential expression for microarray data from genes and miRNAs^24^. In addition, it allows us to standardize the data before performing the analysis. In this context, the Tidyverse package^25^ is used for the efficient loading of large data files.
- **Differential methylation analysis**. Minfi from Bioconductor is used to analyse Illumina DNA methylation arrays^26^. We use Minfi to standarize the data before performing analysis. IlluminaHumanMethylation450kanno.ilmn12.hg19^27^ and IlluminaHumanMethylation450kmanifest^28^ packages are used to annotate each probe with its region (1st exon, 5′ UTR, etc) and type (CpG island, OpenSea,…) in the genome. To perform the differential methylation analysis we also use Limma as a tool for contrasting differences between the two groups.
- **Correlation analysis between microbiome and methylome**. For this analysis, we use the corrplot package^29^. This method returns a table with the selected bacterial genus and genes and the correlation value between their abundance and methylation values. With these results, we use the FactoMineR and Factoextra packages for principal component analysis (PCA) and their visualization, and we also use the cluster^30^ and ape^31^ packages for hierarchical clustering.

### External resources

To enrich the results of the analysis, ColPortal integrates the following external resources:

- **Human Phenotype Ontology (HPO)**. This is an ontology whose main objective is to offer a common vocabulary for the annotation of genes and proteins^32^. HPO is used to link human phenotypic abnormalities with the genes/proteins that cause them or have some kind of influence on them.
- **KEGG**. This is a set of databases to perform functional analysis of genes^33^. In the context of ColPortal, this database will allow us to know the metabolic pathways in which a gene or protein participates.
- **MirTarBase**. This is a database that stores the results of experiments that have been performed to validate the interaction between miRNA and its targets^34^. This tool classifies evidence as strong and weak depending on the type of validation experiment performed.

### Patients and tumour samples

In order to have a wide representation of different histological subtypes of CRC, a previously described series enriched in less frequent CRCs was included in this platform^9,35^. CCs were diagnosed as previously described^36^; SACs were diagnosed on the basis of criteria proposed by Mäkinen *et al*. (epithelial serrations, clear or eosinophilic cytoplasm, abundant cytoplasm, vesicular nuclei, absence of or less than 10% necrosis of the total surface area, mucin production, and cell balls and papillary rods in mucinous areas of a tumour)^8^ and hmMSI-Hs according to prior established criteria (mucinous, signet-ring cell and medullary carcinoma, tumour infiltrating and peritumoural lymphocytes, “Crohn-like” inflammatory response, poor differentiation, tumour heterogeneity, and “pushing” tumour border)^5^. None of the hmMSI-H showed serrated morphology. The study cases consisted of a cohort of 48 SACs, 41 CCs 15 hMSI-H and 58 colorectal polyps retrieved from the Santa Lucia University Hospital, Cartagena, and 18 SACs and 9 CCs from Oulu University Hospital, Oulu, Finland. From these subjects, data from 61 adjacent normal mucosa samples were also included. From all the cases, multiomic data was obtained from frozen tissue specimens. Overall survival (OS) and disease free survival (DFS) were calculated in months as previously described^9,11^. The study was approved by the Hospital Ethics Committee and was carried out in accordance with the ethical standards laid down in the 1964 Declaration of Helsinki and its later amendments. Written informed consent was obtained from all patients. In order to diminish the bias of tumor heterogeneity all samples for DNA- and RNA-based high throughput techniques were taken from the center of the tumor after performing a staining from the frozen tissue block to ensure that no necrotic areas were included.

### Preparation of digital histological slides

Hematoxylin eosin histological preparations were obtained from selected tumor paraffin-embedded blocks showing tumor invasive front and representative morphological features. These slides were digitized at x20 (1.5 GB mean size) using an Aperio AT2 scanner. Digital images are accessible through Clinical cases/List view.

The size of the generated images is between 500 MB and 3 GB. If we upload directly the original images it would be unfeasible for users to see them from the portal. They would have to download them, and install software to see them correctly. We avoid such actions by zooming images using Libvips (https://jcupitt.github.io/libvips/) and the web viewer OpenSeadragon https://openseadragon.github.io/. These technologies have been applied in similar previous works^37–39^.

### DNA extraction

A volume of approximately 10 mm^3^ was extracted from each frozen tissue using the disposable sterile biopsy punch. DNA was extracted following the manufacturer’s instructions (QIAGEN, Hilden, Germany). Briefly, tissue was disrupted and homogenized in ATL buffer using a Tissueruptor (QIAGEN) incubated with proteinase K, and the homogenate was subjected to automatic DNA extraction using the Qiacube equipment and the QiaAmp DNA Mini Kit (Cat No: 51306), both provided by QIAGEN.

### Oncogene mutation testing

These methods are expanded versions of descriptions in our related work^35^. DNA samples were diluted to 5 ng/*μ*l concentration and subjected to allelic discrimination using TaqMan probes for BRAF V600E detection and those cases with no V600E mutation were directly sequenced for BRAF exon 15 as described previously^35^. KRAS mutations at codons 12 and 13 were determined by denaturing high-performance liquid chromatography (dHPLC). A fragment of 92 bp was amplified in a 25 *μ*l volume containing 2 mM MgCl2, 1 mM dNTPs, 10% DMSO and 2U of TaqGold polymerase (Applied Biosystems, Foster City, CA), and 1 *μ*M of primers KRASf and KRASr. To improve the detection of sequence changes in the amplified product, a Guanine-Cysteine clamp was anchored to the 5′ end of the forward primer. The reverse primer contained the M13 Universal sequence to facilitate the subsequent sequencing. Polymerase chain reaction (PCR) cycling conditions were as follows: 9 min 95 °C then 10 cycles of 15 sec at 95 °C, 15 sec at 65 °C and 15 sec at 72 °C followed by 45 cycles of 15 sec at 95 °C, 15 sec at 60 °C and 15 sec at 72 °C. Before dHPLC analysis, PCR products were heated to 95 °C for 10 min and then slowly cooled to room temperature to allow heteroduplex formation. Five microliters of the PCR product were then injected into a preheated reverse-phase column (Helix DVB, Varian Analytical Instruments, 2700 Mitchell Drive, CA) equilibrated by triethylammonium acetate (TEAA) 0.1 M in a Helix ProStar dHPLC instrument (Varian Analytical Instruments, 2700 Mitchell Drive, CA) as previously described^5^. All cases with a curve profile different than KRAS native were confirmed by sequencing using M13 universal primer. The mutation status of exons 9 and 20 in the PI3KCA gene was determined by direct sequencing after a nested-PCR. External and internal PCR were performed in a total volume of 20 *μ*l containing 0.2 mM dNTPs, 2 mM MgCl2 0.025 U/*μ*l GoTaq Hot Start Polymerase (ref: M5001, Promega, Madison, WI) and 0.5 *μ*M of each primer. In the first PCR, 2 *μ*l of DNA template were used with the primers PI542-5EF and PI542-5ER for the exon 9 and with PI1047EF and PI1047ER for the exon 20. Nested PCR was performed using 2 *μ*l of the first PCR and the following primers: PI542-5IF and PI542-5IR for exon 9 and PI1047IF and PI1047IR for exon 20. According to the manufacturer’s instructions, amplicons were purified using the QiAquick 96 PCR purification kit (ref: 28181, Qiagen, Hilden, Germany) and were subsequently subjected to direct sequencing using the primer PI542-5IF for exon 9 and the PI1047seq. Primer sequences are provided in Table 1.

**Table 1:** Primer sequences.

Gene	Primer name	Primer sequence (5′-3′)
**KRAS**	KRASf	TTATAAGGCCTGCTGAAAATGACTGAA
	5′KRASf clamp	CGCCCGCCGCGCCCCGCGCCCGTCCCGCCGCCCCCGCCCCC
	KRASr	TGAATTAGCTGTATCGTCAAGGCACT
**BRAF**	BRAF-51F TaqMan	CTACTGTTTTCCTTTACTTACTACACCTCAGA
	BRAF-176R TaqMan	ATCCAGACAACTGTTCAAACTGATG
	BRAFWT probe	FAM-TAGCTACAGaGAAATC
	BRAFmut probe	VIC-CTAGCTACAGtGAAATC
	BRAFFs	TGCTTGCTCTGATAGGAAAATG
	BRAFRs	CCACAAAATGGATCCAGACA
	BRAFseq	TGATAGGAAAATGAGATCTAC
**PIK3CA**	PI542-5EF	TGCTTTTTCTGTAAATCATCTGTGA
	PI542-5ER	TGCTGAGATCAGCCAAATTC
	PI1047EF	CATTTGCTCCAAACTGACCA
	PI1047ER	GGTCTTTGCCTGCTGAGAGT
	PI542-5IF	GCTAGAGACAATGAATTAAGGGAAA
	PI542-5IR	AAGAAAAAGAAACAGAGAATCTCCA
	PI1047IF	TATTCGACAGCATGCCAATC
	PI1047IR	TGTGTGGAAGATCCAATCCA
	PI1047seq	TTTTGATGACATTGCATACA

### Microsatellite instability and CpG island methylation phenotype (CIMP)

MSI was evaluated, as previously described^35^, using the kit MSI Analysis System, version 1.2 provided by Promega (MI) according to the manufacturer’s instructions. The MSI Analysis System includes fluorescence-labeled primers for co-amplification of seven markers including five mononucleotide repeat markers (BAT-25, BAT-26, NR-21, NR-24 and MONO-27) and two pentanucleotide ones (Penta C and Penta D). The mononucleotide markers are used for MSI determination and the pentanucleotides to detect potential sample mixups and/or contamination. Internal lane size standards are added to the PCR samples to ensure accurate sizing of alleles and to adjust for run-to-run variation.

PCR conditions were 9 min at 95 °C followed by 10 cycles of 30 sec at 94 °C, 45 sec at 60 °C and 30 sec. 72 °C and 35 cycles of 30 sec. 94 °C, 45 sec at 58 °C and 45 sec at 72 °C with an extra extension time of 60 min at 72 °C. One microliter of the PCR product, 0.5 *μ*l of GeneScan500LIZ (Applied Biosystems, Foster City, CA) and 8.5 *μ*l of formamide were analysed in a 3130 Genetic Analyzer (Applied Biosystems).

The cases were categorized as MSI-H or microsatellite stable (MSS)/low-level MSI (MSI-L) according to the NCI criteria^40^.

#### CpG island methylation phenotype (CIMP) assessment

A hundred nanograms of DNA were denatured in a total volume of 5 ml Tris-EDTA buffer, and further performed as recommended by the supplier (MRC-Holland, Amsterdam, the Netherlands). Methylation-specific multiplex ligation-dependent probe amplification (MS-MLPA) is a method for the simultaneous detection of methylation at eight genes firmly associated with CIMP (CACNA1G, IGF2, NEUROG1, RUNX3, SOCS1, CDKN2A, MLH1, and CRABP1) in one reaction^41^. In short, a mixture of probe-mix (ME042-B1 CIMP) and buffer were added to the denatured DNA, and probes were allowed to hybridize to the DNA at 60 °C for 16 hours. Each sample was divided into two tubes, in which one half was ligated, and the other was ligated and digested using the methylation-sensitive restriction enzyme HhaI. Both samples were subsequently subjected to a PCR reaction using a thermal cycler (GeneAmp 2700, Applied Biosystems, Foster City, CA, USA), and fragment analysis performed on a capillary sequencer (ABI 3130xl, Applied Biosystems, Foster City, CA, USA). DNA from normal colonic mucosa was used as normal reference. The output from the analysis, after inter- and intrasample normalization, is a percentage of methylation in the sample. Partially methylated genes were considered as methylated. The Ogino and Weisenberger criteria for CIMP status assessment were applied and compared (CIMP(O) and CIMP(W), respectively)^42,43^ considering only 5 genes from the panel (CACNA1G, IGF2, NEUROG1, RUNX3 y SOCS1), the difference between no-CIMP and high-CIMP being ≤3 and >3 methylated genes, respectively.

### Bisulfite treatment and DNA methylome assay

These methods are expanded versions of descriptions in our related work^12,13^. HumanMethylation450K BeadChip (Illumina, Inc., San Diego, CA), using Infinium HD Methylation assay for genome-wide DNA methylation screening, was employed. In brief, genomic DNA (1000 ng) from each sample was bisulfite converted with the EZ DNA Methylation Kit (Zymo Research, Orange, CA) according to the manufacturer’s recommendations. Bisulfite-treated DNA was isothermally amplified at 37 °C (20–24 h), and the DNA product was fragmented by an endpoint enzymatic process, then precipitated, resuspended, applied to an Infinium Human Methylation450K BeadChip (Illumina, San Diego, CA, USA) and hybridized at 48 °C (16–24 h). The fluorescently stained chip was imaged by the Illumina i-SCAN and Illumina’s Genome Studio program (Methylation Module) was used to analyse BeadArray data to assign site-specific DNA methylation *β* values to each CpG site.

### Microbiota massive genome sequencing

DNA from tumoral and normal adjacent mucosa was extracted as described above. The hypervariable region V3-V4 of the bacterial 16s rRNA gene was amplified using key-tagged eubacterial primers^44^. Illumina adapter overhang nucleotide sequences are added to the gene-specific sequences. The full length primer sequences to follow the protocol targeting this region are:

- 16S Amplicon PCR Forward Primer = 5′: TCGTCGGCAGCGTCAGATGTGTATAAGAGACAGCCTACGGGNGGCWGCAG
- 16S Amplicon PCR Reverse Primer = 5′: GTCTCGTGGGCTCGGAGATGTGTATAAGAGACAGGACTACHVGGGTATCTAATCC

Amplicons (460 bp) were sequenced with a MiSeq Illumina Platform, following Illumina’s handbook 16S Metagenomic Sequencing Library Preparation. The MiSeq run output was approximately >20 million reads which can generate >100,000 reads per sample, commonly recognized as sufficient for metagenomic surveys.

### RNA extraction

A volume of approximately 10 mm^3^ was extracted from each frozen tissue using the disposable sterile biopsy punch Acupunch 2 mm (AcudermInc, Lauderdale, FL, USA) after confirming in a Diff-Quick stain the tumor area to extract. Tissue was disrupted and homogenized in 700 *μ*l of Qiazol (Qiagen ref:1023537) using a Tissueruptor by Qiagen for 20 seconds. The homogenate was incubated at room temperature for five minutes. After adding 140 *μ*l of chloroform and centrifuging at 12,000 × g for 15 minutes at 4 °C, 350 *μ*l of the aqueous phase was subjected to automatic total RNA extraction using the Qiacube equipment and the miRNeasy Mini Kit (ref:217004), both provided by Qiagen.

### mRNA microarray assay

These methods are expanded versions of descriptions in our related work^11,45^. Total RNA was quantified by spectrometry (NanoDrop ND1000, NanoDrop Technologies, Wilminton, DE) and fragment size distribution was analysed by RNA 6000 Pico Bioanalyzer assay (Agilent Technologies, Palo Alto, CA). RNA (150 ng) was concentrated in a SpeedVac to a working dilution and used to produce cyanine 3-CTP-labeled cRNA using the Low Input Quick Amp Labeling Kit, One-Color (Agilent p/n 5190-2305) according to the “One-Color Microarray-Based Gene Expression Analysis” protocol Version 6.0 (Agilent p/n G4140-90040). This method uses T7 RNA polymerase which simultaneously amplifies the target material and incorporates cyanine 3-labeled-CTP. A 2,000 ng cRNA product was hybridized with a Whole Human Genome Oligo Microarray Kit (Agilent p/n G2519F-014850) containing 41,000 unique human genes and transcripts. Arrays were scanned in an Agilent Microarray Scanner (Agilent G2565BA) according to the manufacturer’s protocol and data extracted using Agilent Feature Extraction Software 10.7.1 following the Agilent grid template 014850_D_F_20100430 protocol GE1_107_Sep09 and the QC Metric Set GE1_QCMT_Sep09.

### miRNA microarray assay

miRNA microarray profiling was conducted using Affymetrix GeneChip miRNA 3.0 arrays (Affymetrix, Inc., Santa Clara, CA, USA) containing 5,607 probe sets for human pre-miRNA, miRNAs, small nuclear RNA, and small Cajal body-specific RNA. These Affymetrix miRNA arrays provide 100% coverage of the miRNAs in the miRBase (version 17; http://www.mirbase.org). Probe sets that were deleted in a more recent version of miRBase (version 21) were excluded from the analysis. All steps of the procedure were performed according to the Affymetrix standardized protocol for miRNA 3.0 arrays.

### Strengths and limitations

Based on the principle that the histology is a complex manifestation of genetic and epigenetic changes, the main goal of the included series is to provide a representation of different histological subtypes of colorectal cancers with their associated “omic” profiles. Those profiles have been obtained enriching certain subtypes, such as serrated adenocarcinoma and colorectal carcinomas, with histological features of microsatellite instability. Therefore, it does not represent the general frequency of these subtypes found amongst non-selected patients with colorectal carcinoma. The benefits of ColPortal for clinicians is the possibility of correlating not very common CRC subtypes with clinical features such as tumor presentation and prognosis; for pathologists it allows to associate histological characteristic of CRC with particular molecular changes. Finally, researchers can generate proof of concepts and establish relationships amongst genetics and epigenetics features at different levels (e.g. microbiota and microtranscriptomics; microbiota and methylomics, etc). The main advantage compared to the TCGA database is that ColPortal allows the integration of microbiota and methylome data in cancer and precursor lesions. On the other hand, the main limitation of ColPortal is the number of cases included for each platform. Nevertheless, once the frame and data acquisition has been created new cases and datasets will be incorporated. Another limitation of the portal is the absence of proteomic data especially post-translational modifications which are important elements affecting protein functionality and therefore the tumor histology and biology.

## Data Records

In Table 2 we detail the types of datasets and their number of cases/samples used in the analyses. Next, we describe this data and their availability:

- **Methylome**. Raw data from HumanMethylation450K BeadChip (Illumina, Inc., San Diego, CA) and Infinium HD Methylation assay for genome-wide DNA methylation screening providing information of methylation status of 450,000 CpG sites from 118 samples^46,47^.
- **Microbiome**. Illumina Next-generation-sequence of bacterial 16S rRNA (V3-V4) from 88 samples^48–50^.
- **Transcriptome**. Agilent One-color microarray-based gene expression analysis protocol version 6.0 (Agilent p/n G4140-90040) from 58 samples^51,52^.
- **MicroTranscriptome**. Agilent One-color microarray-based gene expression analysis protocol version 6.0 (Agilent p/n G44710C-021827) from 26 samples^53,54^.
- **Images**. In^55^ 3 original images from hematoxylin eosin histological preparations corresponding to colorectal cancer tissues are available. ColPortal has available 48 zooming tissue images joined to tumoral clinical cases.
- **Clinical cases**. In^56^ 253 cases with clinical information associated with the previous datasets are available.

**Table 2:** Types of datasets available and their number of cases/samples.

Datasets	Cases/Samples
Clinical cases	253
Tissue Images	48
Methylome	118
Microbiome	88
Transcriptome	58
MicroTranscriptome	26

## Technical Validation

The technical validation of this cohort includes the use of ColPortal as an anonymous user. A glossary of the terminology used in ColPortal is shown in Table 3.

**Table 3:** Glossary of terms.

Term	Description
3′UTR	3′ untranslated region
1stExon	First exon of a gene
5′UTR	5′ untranslated region
TSS200	Up to 200 bp from transcription start site
Body	Region within gene
TSS1500	Up to 1500 bp from transcription start site
Island	Region >200 bp in length with GC percent >50% and observed/expected ratio >0.6 for CG
[N/S]_shore	Regions up to 2 kb from CpG island (upstream or downstream)
[N/S]_shelf	Regions up to 2–4 kb from CpG island (upstream or downstream)
OpenSea	Rest of genome excluding CpG islands, shores and shelves
CIMP Ogino	CpG Island Methylation Phenotype (evaluation method following criteria by Ogino *et al*.)
CIMP Weisemberg	CpG Island Methylation Phenotype (evaluation method following criteria by Weisemberg *et al*.)
Disease Status	disease status of sample. Possible values: tumoral, polyp and normal
Grade	histological tumor grade according to WHO
MSI status	Microsatellite Instability status
TNM	staging system for tumors
T stage	describes the tumor size and any spread of cancer into nearby tissue
N stage	describes spread of cancer to nearby lymph nodes
M stage	describe metastasis
Tumor budding	describes the presence of clusters of tumoral cells detaching from invasive margin of main tumor
TB Grade	tumor budding grade
Type	hystological type of the tumor

### Clinical cases

**Fig. 2:**
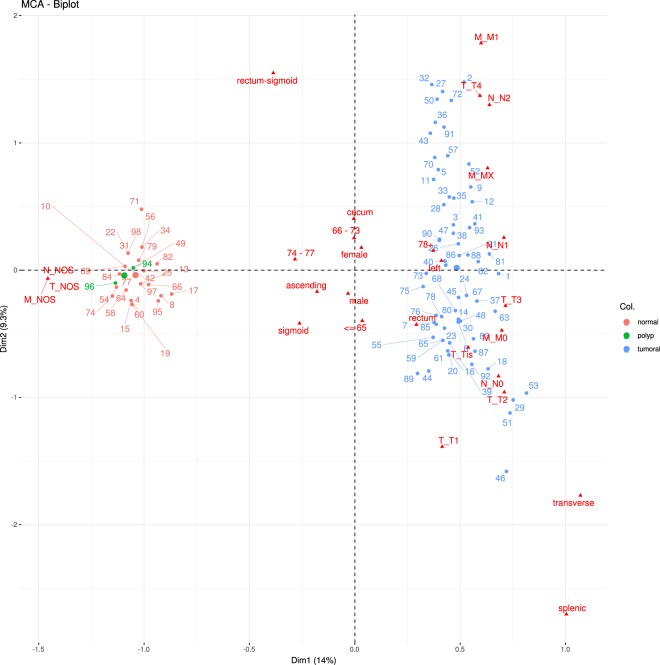
MCA analysis of clinical cases classified by disease status (normal, polyp, tumoral), using the variables age, sex, general localization and T, N and M staging.

First of all, we will validate whether the data referring to clinical cases are capable of correctly classifying the different types of samples from the portal. To perform this task, from the clinical cases section, the user can perform an MCA analysis selecting the classification variables (*Classes*) and the variables that will be used for generating that classification (*Variables*). Figure 2 shows the result of an MCA analysis^22^ with the tumoral status classification (variable disease status as class) using age group, sex, general localization and TNM staging (age, sex, general localization, T stage, N stage, M stageas variables). In this case, the two groups are clearly separated using data from the clinical cases.

### Gene regulation

ColPortal allows the generation of customizable analyses over RNA and miRNA data from Transcriptome and MicroTranscriptome sections. In this validation case, we have used the preloaded methylome information, where we have differential methylation data between normal and tumoral samples. The cohort selection includes normal and tumoral samples with RNA and Methylome data. In this case we have 2 normal cases and 24 tumoral cases. We use the Limma package^24^ to perform the differential expression analysis. The analysis returns 5,309 differentially expressed genes with adjusted p-values of less than 0.05. The data can be filtered by gene name, KEGG pathway^33^ or by human phenotype (from Human Phenotype Ontology^32^) and ordered using different criteria.

**Fig. 3:**
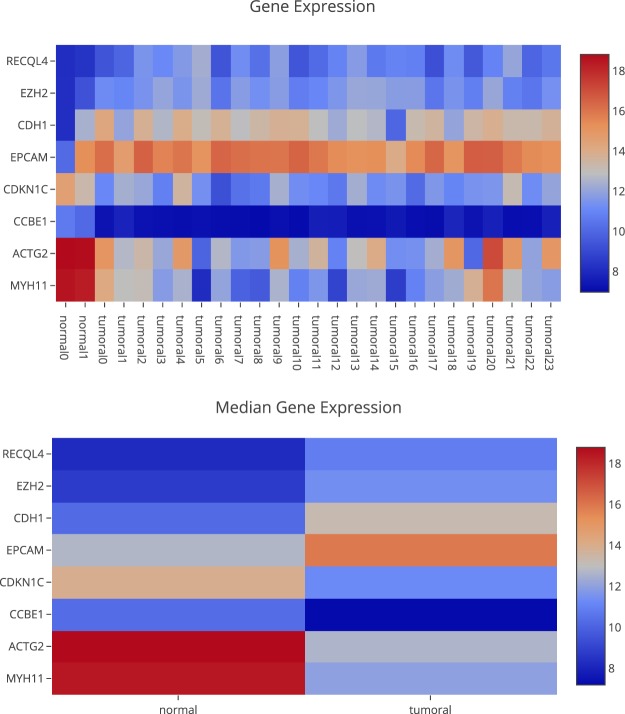
Differential gene expression for genes filtered by human phenotypes related with cancer, colon, rectal and rectum and with highest absolute value for log of fold change in the 26 preloaded clinical cases.

For this example, we first filter the genes using keywords such as cancer, colon, rectal and rectum for human phenotypes. Then we ordered the genes by absolute value for log of fold change and visualized (pressing the eye button) the genes with the highest value. As a result we obtained the gene expression heatmaps for the resulting 8 genes and the median methylation for each gene. Figure 3 shows the gene expression.

**Fig. 4:**
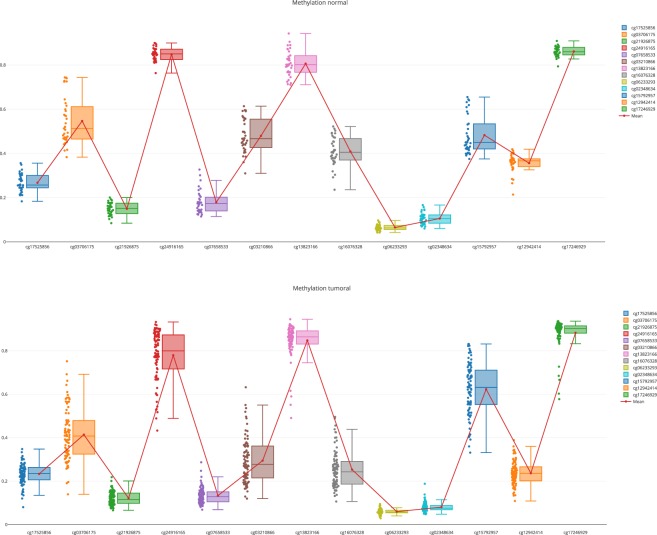
Methylation pattern for the gene EPCAM.

**Fig. 5:**
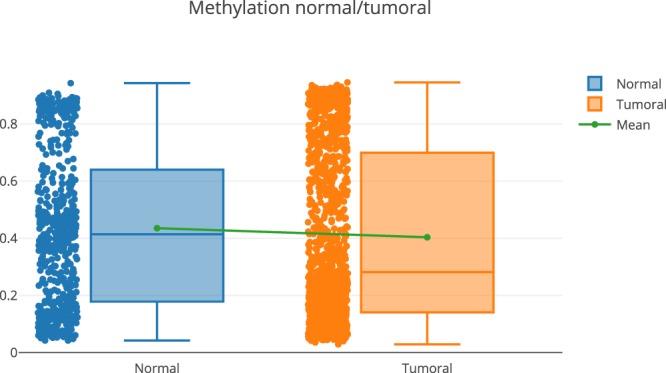
Methylation pattern (normal vs tumoral) for the gene EPCAM.
- A differential expression analysis of miRNAs and check if those genes are regulated by this molecular mechanism. This can be done in the “MicroTranscriptome” section, where you can filter by different features. Figure 6 shows the hsa-miR-221 expression. Our platform uses data from MirTarBase^34^ to know the targets validated in laboratories and CDKN1C is one of them. EPCAM cannot be validated because methylation and miRNAs do not only regulate gene expression.

The results show that the expression of some genes are inversely correlated with methylation values, which validates the quality of data. However, the correlation with methylation for genes such as CDKN1C and EPCAM is not clear. For these cases, the user has two options:

- A more detailed exploration of the methylation pattern of this gene. This can be done in the “Methylome section”, “Methylome Gene Visualization” tab, filtering by gene regions, CpG islands, transcription start site, etc. Figure 4 shows the result of filtering by gene EPCAM. The user can also see the comparison between normal and tumoral (see Fig. 5).

**Fig. 6:**
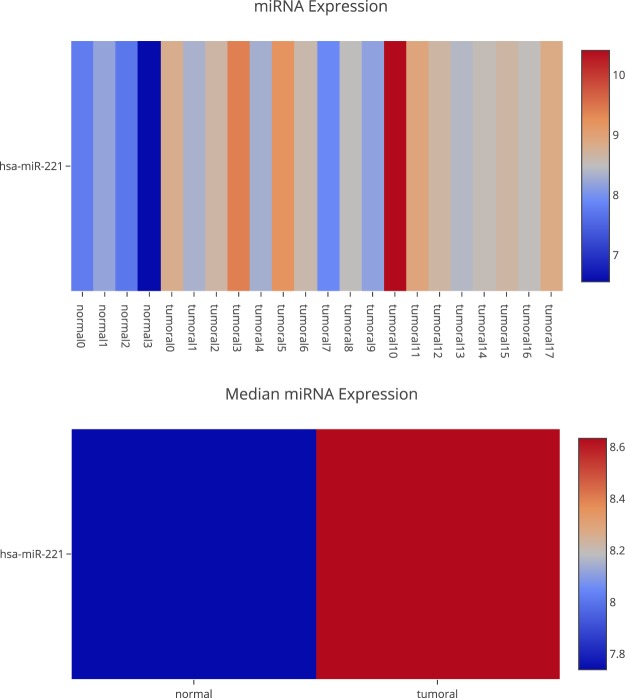
Differential miRNA expression for the miRNA hsa-miR-221.

### Methylome and microbiome interaction

**Fig. 7:**
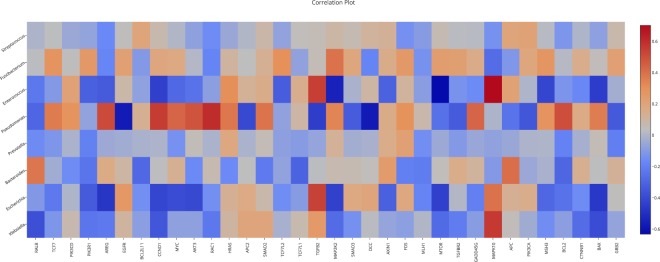
Correlation plot between Microbiome and Methylome.

In this case, we use the Microbiome section for selecting microbiome genus and methylated genes using the following filters: (1) genus abundance greater than 20 and (2) genes with an absolute value of log of fold change greater than 1 in the colorectal cancer pathway. With this filter we recovered 8 genus and 34 genes. Figure 7 shows the correlation plot between microbiome and methylome using the previous filtered data.

**Fig. 8:**
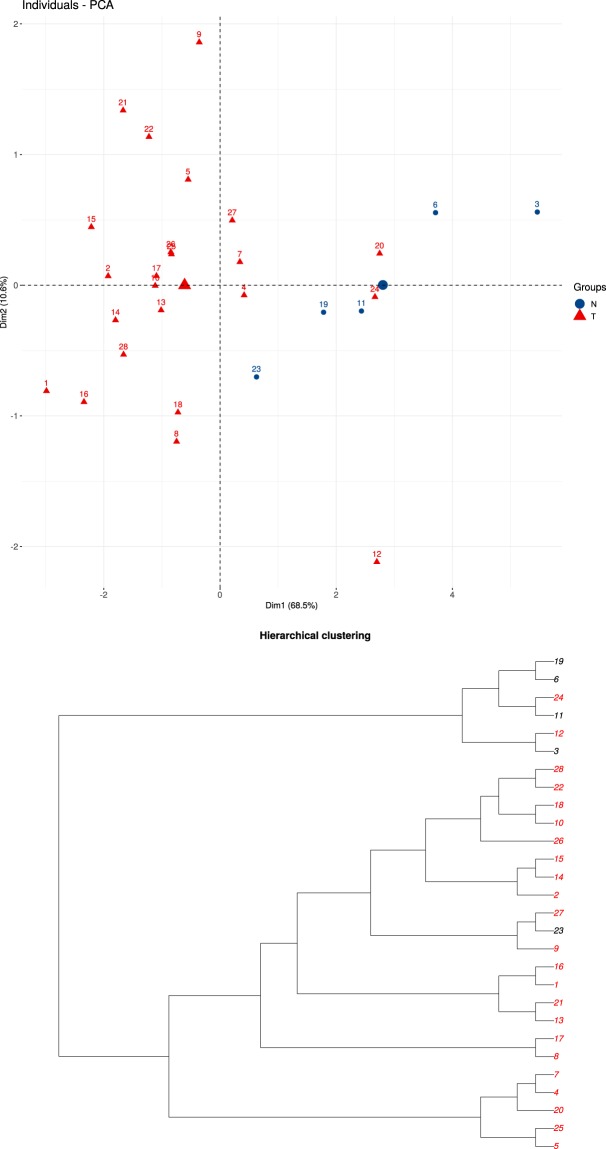
PCA and Hierarchical Clustering between Microbiome and Methylome.

Now, the user can filter correlated genus and gene and perform a Principal Component Analysis^22^ and a Hierarchical Clustering^30^ analysis. Figure 8 shows that the pseudomonas genus (the genus with more correlations) allows us to correctly separate normal and tumour samples using also their methylation values. In^57^ we found a study that describes the prevalence of a pseudomonas species in patients with cancer.

## Usage Notes

ColPortal allows to analyse in an integrated way all the data of this cohort. However, if users want to reuse the data for other studies in Figshare all the data and R scripts are available to perform the analyses^46,48,49,51,53,56,58^. All the omic raw data can be linked with the metadata from clinical cases using the field labeled *Id*.

## Data Availability

These are the scripts^58^ used in the different analyses: • mRNA expression script. This script reads preprocessed and normalized expression data and makes a differential expression (DE) analysis between the samples defined in the parameter $\#\#\#URL\#\#\#$. As a result, a table of DE genes is generated. A second table with the normalized gene expression values per sample is also generated. • miRNA expression script. This script reads preprocessed and normalized miRNA expression data and makes a differential expression (DE) analysis between the samples defined in parameter$\#\#\#URL\#\#\#$. As a result, a table of DE miRNA is generated. Likewise, a second table with the normalized gene expression values per sample is generated. • Microbiome correlation script. This script generates a correlation plot between microbial abundance and methylation data from selected DM genes obtained from samples previously filtered. $\#\#\#microbioma\,Genes-Methylated\#\#\#$ is a table whose variables are the microbiome data and the methylation levels of the genes for the selected samples. An extra variable called “class” contains the labels of the classes. It is also necessary to know the number of microbiome variables (###*numgenus*###) and the number of genes (###*numGenes*###). • Microbiome PCA script. This script generates a plot resulting from a principal components analysis (PCA) using the methylation values of the selected genes in the filtered samples, and the selected genus(s) (or families, or species). The color distinguishes the different groups of samples. • Methylation script. This script performs a differential methylation (DM) analysis using raw data from Illumina microarrays. The results are a DM gene table and the methylation values (beta values) of each sample. • MCA plot script. This performs a multiple correspondence analysis with the selected variables. The result is a graph with the relations between categories. All scripts are developed in R^59^ and they are used as templates, which are instantiated using the values defined by the user. The following R packages have been used: limma^24^, tidyverse^25^, FactoMineR^22^, factoextra^23^, corrplot^29^, minfi^26^, IlluminaHumanMethylation450kanno.ilmn12.hg19^27^, IlluminaHumanMethylation450kmanifest^28^, cluster^30^ and ape^31^.
